# *In Vitro* Anti-oxidant Effect of Vitamin E on Oxidative Stress Induced due to Pesticides in Rat Erythrocytes

**DOI:** 10.4103/0971-6580.75871

**Published:** 2011

**Authors:** Ronika Saxena, Poonam Garg, D. K. Jain

**Affiliations:** Department of Chemistry, Bareilly College, Bareilly - 243 005, Uttar Pradesh, India; 1 Department of Zoology, Bareilly College, Bareilly - 243 005, Uttar Pradesh, India

**Keywords:** Catalase, chlorphyrifos, endosulfan, erythrocytes, lipid peroxidation, superoxide dismutase, vitamin E

## Abstract

An attempt was made to study the antioxidant property of vitamin E in endosulfan and chlorpyrifos toxicity. Erythrocytes were collected from healthy rats and exposed to 1 ppm endosulfan and chlorpyrifos pesticides individually and also along with vitamin E treatment. Results showed that endosulfan was more toxic in comparison of chlorpyrifos. Activities of superoxide dismutase and catalase were significantly decreased, while lipid peroxidation and glutathione-*S*-transfarase were increased in comparison to the control values. The results of the present study suggest that vitamin E acts as an effective antioxidant for endosulfan and chlorpyrifos pesticide toxicity, in reducing oxidative stress burden.

## INTRODUCTION

Advent of agricultural and industrial revolution in India has added many pollutants in the environment, which are potentially hazardous, out of which some may be toxic, inflammable, explosive or corrosive. Of the various pollutants present in the hydrosphere and lithosphere, heavy metals and pesticides are toxic to livestock as well as human beings. However, not all the pesticides are actually toxic for humans or other non-target species.[1]

Endosulfan (6, 7, 8, 9, 10, 10 -hexachloro- 1, 5, 5a, 6, 9, 9a -methano -2, 4, 3 -benzodioxathiepin- 3 -oxide) is an organochlorine insecticide and acaricide, and acts as a contact poison in a wide variety of insects and mites.[2] It is being easily absorbed by the stomach, lungs and through the skin, which means that all routes of exposure can pose a hazard. It enhances the effect of estrogens and acts as an endocrine disruptor, causing reproductive and developmental damage in animals and humans, as well as cancer. Recent studies indicate that pesticide intoxication produces oxidative stress by the generation of free radicals and by inducing tissue lipid peroxidation in mammals and other organisms.[3] Hincal *et al*,.[4] reported the oxidative stress inducing effects of endosulfan, with an increase of lipid peroxidation and a significant alteration in glutathione (GSH) redox cycle in cerebral and hepatic tissues of rats.

The organophosphorus (OP) insecticide, chlorpyrifos (*O*,*O*-diethyl O-3, 5, 6-trichloro-2-pyridyl-phosphorothioate), is widely used for a variety of agricultural and human health applications. OPs produce a wide range of toxicity in mammals by inhibiting acetylcholinesterase (AChE), and the consequent accumulation of the neurotransmitter acetylcholine (ACh) in synaptic junction leads to excessive stimulation of postsynaptic cells causing cholinergic toxicity.[5] In fact, one of the molecular mechanisms of the toxicity of some pesticides seems to be lipid peroxidation. As a consequence, these compounds can disturb the biochemical and physiological functions of the red blood cells (RBCs).[6] The susceptibility of RBC to oxidative damage is due to the presence of polyunsaturated fatty acid, heme iron and oxygen, which may produce oxidative changes in RBC.[7]

Major contributors to non-enzymatic protection against lipid peroxidation are vitamin E and vitamin C, well-known free radical scavengers.[8] Vitamin E is a lipid soluble, chain-breaking antioxidant playing a major protective role against oxidative stress and prevents the production of lipid peroxides by scavenging free radicals in biological membranes. Some investigators reported that administering Vitamin E may be useful in controlling the toxic effect of insecticides and chemicals.[9] Keeping these points in mind, the present study was planned to establish the antioxidant role of vitamin E on oxidative stress induced in rat RBCs by pesticides.

## MATERIALS AND METHODS

Ten male healthy rats weighing about 100–200 g were used in the study. About 3 ml peripheral blood was obtained from ocular vein/heart puncture of rats, using ethylenediaminetetraacetic acid (EDTA)-sodium salt as the anticoagulant for assays. Blood was centrifuged at 2000 rpm for 10 min. Plasma and buffy coat was removed. Subsequently, the cells were washed three times with phosphate buffered saline (PBS), pH 7.2. The final red cell suspension was taken in test tubes for chlorpyrifos, endosulfan and vitamin E treatment, each in triplicate set. Pesticides and vitamin E (chlorphyrifos, endosulfan, vitamin E, chlorphyrifos and vitamin E, and endosulfan and vitamin E) were dissolved in dimethyl sulfonic acid (DMSO) and the solution was made up 100 ppm stock solution of each group, respectively. The above combinations were mixed and the desired 1 ppm concentration was made in all groups (DMSO 5% of total volume and test was performed in triplicate set). Also, 5% DMSO was dissolved/mixed in control group. The tubes were incubated for 3 hours at 37°C in a shaking water bath. At the end of incubation, the tubes were removed and subjected to biochemical analysis.

### Biochemical analysis

#### Chemicals for analysis

Alpha endosulfan (100%) and chlorpyrifos (100%) were purchased from Accustandard Inc., NH, USA. Tris cacodylic acid, diethylenetriamine-penta-acetic acid (DTPA) (99% pure), nitroblue tetrazolium (98%), pyrogallol (>98%), sodium dodecyl sulfate (>99%), bovine serum albumin (97%), triton X-100 and thiobarbituric acid (99%) were purchased from Sigma Chemicals (USA). Nitric acid (69%), NaH_2_PO_4_ (98%), KH_2_PO_4_ (99.5%), pyridine (99%) and 1-butanol (0.99%) extra pure grade were purchased from Qualigens Chemicals, Mumbai, India.

#### Lipid peroxidation in erythrocytes

The level of lipid peroxides (LPOs) in erythrocyte hemolysate was determined spectrophotometrically following the method of Placer *et al*.[10] Lipid peroxidation was calculated using 1.56 × 10^5^ as extinction coefficient[11] to express the value in nanomoles of malondialdehyde (MDA) per milliliter of hemolysate. Hemoglobin (Hb) in hemolysate was estimated spectrophotometrically by cyanomethemoglobin method[12] and LPO level in the erythrocytes was expressed as nanomoles of MDA per mg of hemoglobin.

#### Antioxidant enzymes

The erythrocyte homogenate was used for analysis of antioxidant enzymes after suitable dilution. Activity of superoxide dismutase (SOD) was measured in the 10% RBC hemolysate, following the method of Marklund and Marklund[13] with certain modifications suggested by Minami and Yoshikawa[14]. Each unit of SOD activity is defined as the quantity of enzyme that inhibits auto-oxidation of pyrogallol by 50% under suitable experimental conditions. Catalase (CAT) activity in RBC hemolysate was estimated spectrophotometrically at 240 nm wavelength after appropriate dilution, following the method of Cohen *et al*.[15] and the values were expressed in units per milligram of hemoglobin for erythrocytes. Glutathione-*S*-transferase (GST) activity in RBC hemolysate was estimated spectrophotometrically at 340 nm wavelength as per the method of Habig *et al*.[16] and the values were expressed in units per milligram of hemoglobin for erythrocytes.

### Statistical analysis

The data obtained were statistically analyzed using two-way analysis of variance to find out significance of difference within a group at different periods of observation and Student’s *t*-test to find out the significance between the groups at a particular period of observation.[17]

## RESULTS AND DISCUSSION

Data pertaining to Hb concentration and enzyme activity are presented in Table 1. There was significantly higher (*P*<0.05) Hb level in the endosulfan exposed group and lowest level was found in the control group (without exposure and treatment) erythrocyte cell lysate. The level of LPO in cell lysate was significantly (*P*<0.05) greater in the group exposed to endosulfan and chlorpyrifos, and lowest in the control group. The LPO level in cell lysate reduced significantly (*P*<0.05) in vitamin E treated groups as compared to their respective non-treated pesticide exposed group. The SOD activity was significantly lower in endosulfan and chlorpyrifos exposed groups (18.16±0.44 and 26.14±0.52, respectively). Rats’ erythrocytes exposed to endosulfan and chlorpyrifos at 1 ppm concentration and on treatment with vitamin E showed greater SOD activity compared with non-treated pesticide exposed group. It was with duration treatment (3 hrs) E treatment improved SOD activity with duration treatment in both pesticide groups. However, vitamin E treatment in both pesticide exposures increased CAT activities in erythrocytes. The CAT activity in erythrocytes decreased gradually following both the pesticide exposures. The GST activity in erythrocytes increased significantly (*P*<0.05) in pesticide exposed groups as compared to their respective vitamin E treated group. The GST activity was significantly higher in endosulfan and chlorpyrifos exposed groups (2.16±0.04 and 3.63±0.03, respectively).

**Table 1:** Activities of antioxidant enzymes in erythrocytes of the treatment groups

	Hb (g/dl)	LPO (nmoles MDA/mg of Hb)	SOD (units/mg of Hb)	CAT (units/mg of Hb)	GST (units/mg of Hb)
(*n* = 6)	Mean±SE	Mean±SE	Mean±SE	Mean±SE	Mean±SE
Control	7.33±0.01^a^	8.45±0.00^a^	32.55±0.02^d^	115.02±0.47^f^	1.13±0.00^b^
Chlorphyrifos	7.42±0.02^abc^	9.21±0.03^c^	26.14±0.52^b^	80.00±1.06^c^	3.63±0.03^c^
Endosulfan	7.52±0.04^c^	10.43±0.08^d^	18.16±0.44^a^	46.00±0.73^a^	2.16±0.04^d^
Vitamin E	7.30±0.03^ab^	8.78±0.00^b^	32.08±0.07^d^	109.00±0.37^e^	1.03±0.01^a^
Chlorphyrifos + Vitamin E	7.40±0.06^abc^	8.81±0.06^b^	29.06±0.15^c^	98.02±0.89^d^	1.58±0.02^b^
Endosulfan + Vitamin E	7.45±0.06^bc^	9.10±0.05^c^	25.99±0.10^b^	71.83±0.83^b^	1.23±0.04^c^

Values bearing different superscripts in a row differ significantly (*P*<0.05)

Our studies comprise a part of comparative toxicology studies aimed to identify the biochemical and physiological alterations in RBCs exposed to two different pesticides. The approach is known to help in understanding the mechanisms of toxic action due to xenobiotics.[18]

There are several pathways by which the pesticide is thought to induce oxidative stress. It inhibits the mitochondrial electron-transfer chain reaction, leading to accumulation of semi ubiquitous, which enables it to transfer one electron (e^–^) to molecular oxygen to form superoxide radicals.[19] Further, it may also interfere with cellular antioxidant defense system via alteration in activities of antioxidant enzymes, viz., SOD and CAT and status of GSH.[20] LPO level in rat erythrocytes treated with vitamin E was comparable to that of control, suggesting that the pesticides (endosulfan and chlorphyrifos) act as catalysts in the oxidative deterioration of biological macromolecules and this effect could be minimized by treatment with antioxidants.

These indirectly suggest an increased production of oxygen free radicals in erythrocytes. Highly reactive oxygen metabolites, especially hydroxyl radicals, act on unsaturated fatty acids of phospholipid components of membranes to produce MDA, a lipid peroxidation product. Chlorpyrifos has been reported to induce oxidative stress, as shown by enhanced MDA production.[21] The use of vitamin E in conjunction with chlorpyrifos affected such an elevation in the level of MDA, bringing it within the normal limits. The normalization of LPO following vitamin E treatment is very likely due to its antioxidant properties.

Our results revealed that endosulfan and chlorpyrifos caused a statistically significant decrease (*P*<0.05) in SOD activity in rat erythrocytes compared to the control value. Supplementation of vitamin E to endosulfan and chlorpyrifos treated groups of rat erythrocytes normalized the levels of SOD. Treatment with vitamin E alone did not result in significant alteration in SOD activity compared to control treatment. The decrease in the activity of SOD in chlorpyrifos-intoxicated animals may be attributed to the consumption of this enzyme in converting O^2–^ to H_2_O. The dismutation of O^2–^ to H_2_O is catalyzed by SOD which contains both copper and zinc.

In comparison to the control group, the activity of GST was significantly (*P*<0.01) higher in chlorpyrifos treated rat erythrocytes. Considering that GSTs are detoxifying enzymes that catalyze the conjugation of a variety of electrophilic substrates to the thiol group of GSH, producing less toxic forms, the significant increase of GST activity in the rat erythrocytes after exposure to endosulfan and chlorpyrifos may indicate sufficient detoxification of pesticide in rat erythrocytes while the use of vitamin E with pesticide aproaches the control group.

CAT is ubiquitously present in a wide range of aerobic cell types, with the highest activities in mammals being found in liver, kidney and RBCs. Endosulfan and chlorpyrifos caused significant decrease in CAT activity in erythrocytes of rats in this study. In comparison, vitamin E with endosulfan and chlorpyrifos treated erythrocytes maintained the levels of CAT at the normal values.

In conclusion, treatment with vitamin E potentially reduced the free radicals in erythrocytes and ameliorated the oxidative stress as evidenced from lower concentrations of LPOs and GST and higher activities of SOD and CAT in erythrocytes. The efficacy of vitamin E in ameliorating pesticide-induced oxidative stress was higher for chlorpyrifos than for endosulfan.
